# Rapid Ammonia Clearance Using Intensified Continuous Kidney Replacement Therapy in a Neonate With Type 1 Citrullinemia: A Case Report

**DOI:** 10.7759/cureus.117930

**Published:** 2026-10-14

**Authors:** Mirjana Cvetkovic, Kristina Matijas, Svetlana Buljugic, Ana Petrovic, Brankica Spasojevic

**Affiliations:** 1 Faculty of Medicine, University of Belgrade, Belgrade, SRB; 2 Department of Nephrology, University Children’s Hospital, Belgrade, SRB; 3 Intensive Care Unit, University Children’s Hospital, Belgrade, SRB

**Keywords:** case report, citrullinemia type 1, hyperammonemia, kidney replacement therapy, neonate

## Abstract

Neonatal-onset type 1 citrullinemia is an inherited urea cycle disorder in which impaired ammonia detoxification may rapidly lead to severe hyperammonemia requiring extracorporeal support. We report a full-term neonate with severe hyperammonemia (478 µmol/L) successfully treated with intensified continuous kidney replacement therapy (CKRT). Initial conventional CKRT achieved limited ammonia reduction. Escalation to an intensified continuous hemodialysis protocol, incorporating higher blood and dialysate flow rates and a larger hemofilter, resulted in rapid ammonia clearance to 92 µmol/L within 10 hours. Neurological status improved promptly in parallel with the biochemical response, without hemodynamic instability. Transient hypokalemia, hypomagnesemia, and hypophosphatemia were observed and corrected. Genetic analysis confirmed type 1 citrullinemia due to pathogenic variants in the *ASS1* gene. This case demonstrates that intensified CKRT can achieve rapid and effective ammonia removal in neonatal hyperammonemia, even in centers without dedicated neonatal dialysis platforms. Early recognition and timely intensification of CKRT may improve neurological outcomes.

## Introduction

Type 1 citrullinemia is a rare autosomal recessive urea cycle disorder with a prevalence of 1:220,000 births [1]. It is caused by a deficiency of the enzyme argininosuccinate synthetase (ASS), which plays a key role in the third step of the urea cycle by catalyzing the condensation of citrulline and aspartate to form argininosuccinic acid. Enzyme deficiency results in impaired urea biosynthesis and excessive accumulation of citrulline and nitrogen in the form of ammonia, resulting in toxic hyperammonemia [1]. The neonatal form, which represents the most severe phenotype, typically presents within the first days of life with progressive neurological deterioration, lethargy, vomiting, respiratory distress, and encephalopathy [2,3]. Delayed diagnosis and treatment are associated with a high risk of irreversible neurological injury and death [1,3,4,5]. The principal therapeutic goal during acute hyperammonemic crises is prompt reduction of plasma ammonia concentrations to minimize neurological injury [6,7]. In addition to conservative treatment, including disease-specific pharmacological therapy and appropriate nutritional support, extracorporeal blood purification techniques play a pivotal role in achieving high ammonia clearance [1,6,7]. Kidney replacement therapy (KRT), particularly continuous kidney replacement therapy (CKRT), has emerged as an effective treatment modality for neonates with severe hyperammonemia (>400 µmol/L) and clinical signs of encephalopathy or coma [7,8]. To maximize ammonia clearance, intensified CKRT protocols have been developed, incorporating higher blood flow (Qb), dialysate flow (Qd), and replacement fluid (RF) flow rates, as well as hemofilters with membrane surface areas disproportionately larger than the patient's body surface area [2,7].

We report a neonate with life-threatening hyperammonemia caused by type 1 citrullinemia who was successfully treated using an intensified CKRT protocol. This case highlights the practical aspects of delivering intensified CKRT in a center without a dedicated neonatal dialysis platform and demonstrates its effectiveness in achieving rapid ammonia clearance and neurological recovery.

## Case presentation

A male neonate was born by normal vaginal delivery to a primigravida at 39 weeks of gestation after an uneventful pregnancy, with normal anthropometric parameters (body weight 3.3 kg, body length 54 cm) and Apgar scores of 9 and 10. No consanguinity or hereditary or chronic diseases were reported in the family history. From the first day of life, the neonate was exclusively breastfed and was discharged from the maternity hospital after three days. Stools and urine output were normal. Following discharge, he developed poor feeding and recurrent regurgitation and was readmitted on the sixth day of life. Physical examination on admission was unremarkable, but within eight hours of admission, he became progressively lethargic and experienced three episodes of focal seizures. Laboratory evaluation revealed severe hyperammonemia (478 µmol/L). Additional laboratory results are presented in Table 1.

**Table 1:** Laboratory findings at the admission

Variables	Admission
Ammonia (µmol/L)	478
Glucose (mmol/L)	3.7
pH	7.44
Bicarbonate (mmol/L)	17.7
Lactate (mmol/L)	5.2
Sodium (mmol/L)	141
Potassium (mmol/L)	5.6
Ionized calcium (mmol/L)	1.35
Urea nitrogen (mmol/L)	<1.8
Creatinine (µmol/L)	40
Aspartate aminotransferase (U/L)	38
Alanine aminotransferase (U/L)	24
Total/direct bilirubin (µmol/L)	266/15.6
C-reactive protein (mg/L)	11.8

In addition to conservative management with intravenous sodium benzoate and parenteral nutrition providing a high glucose and lipid intake, CKRT was initiated using continuous venovenous hemodiafiltration (CVVHDF) with standard flow settings (Qd/RF ≈ 2,000 mL/h/1.73 m², Qb 5 mL/kg/min) and a Prismaflex HF20 hemofilter (Gambro Industries, Meyzieu, France), with a membrane surface area of 0.2 m². Vascular access was successfully established during the overnight on-call period (5.5-French central venous catheter in the right femoral vein and a 22-gauge intravenous cannula in the left axillary artery), allowing prompt initiation of treatment. The extracorporeal circuit was primed with packed red blood cells, and systemic anticoagulation was maintained with unfractionated heparin. The plasma ammonia concentration decreased to 387 µmol/L after six hours and to 382 µmol/L at the end of treatment (13 hours), when the session was prematurely terminated because of circuit clotting (Figure 1). A second CKRT session, delivered as continuous venovenous hemodialysis (CVVHD), was initiated later the same day with an intensified treatment protocol. After replacement of the vascular access (22-gauge intravenous cannula in the left femoral artery, replacing access to the axillary artery), higher blood and dialysate flow rates could be achieved (Qd ≈ 4,000 mL/h/1.73 m², Qb 8 mL/kg/min) using a Prismaflex ST60 hemofilter (Gambro Industries, Meyzieu, France), with a membrane surface area of 0.6 m². The Prismaflex system (Gambro Industries, Meyzieu, France) was used in both sessions. In the second session, the achieved dialysate flow rate was limited by technical considerations related to vascular access. Plasma ammonia levels declined to 213 µmol/L after four hours and to 92 µmol/L after 10 hours of treatment (Figure 1). Neurological recovery closely followed the decline in plasma ammonia concentrations, with restoration of spontaneous motor activity and the sucking reflex and no further seizures. During treatment, hypokalemia (2.9 mmol/L), hypomagnesemia (0.5 mmol/L), and hypophosphatemia (0.8 mmol/L) developed but were promptly recognized and successfully corrected with intravenous electrolyte supplementation. No hemodynamic instability occurred during either CKRT session. From the fourth hospital day onward, conservative therapy alone maintained stable plasma ammonia concentrations (Figure 1). On the ninth hospital day, the patient was transferred to a tertiary referral center for further management of an inherited metabolic disorder. Metabolic investigations demonstrated markedly elevated plasma citrulline levels (2,889 µmol/L), and genetic analysis identified two heterozygous variants in the ASS1 gene: a pathogenic (Class 1) missense variant, c.1168G>A (p.Gly390Arg), and a likely pathogenic (Class 2) deletion variant, confirming the diagnosis of type 1 citrullinemia. At the most recent follow-up, at seven months of age, the patient was in good general condition, with only a mild delay in motor development, and was undergoing evaluation for liver transplantation.

**Figure 1:**
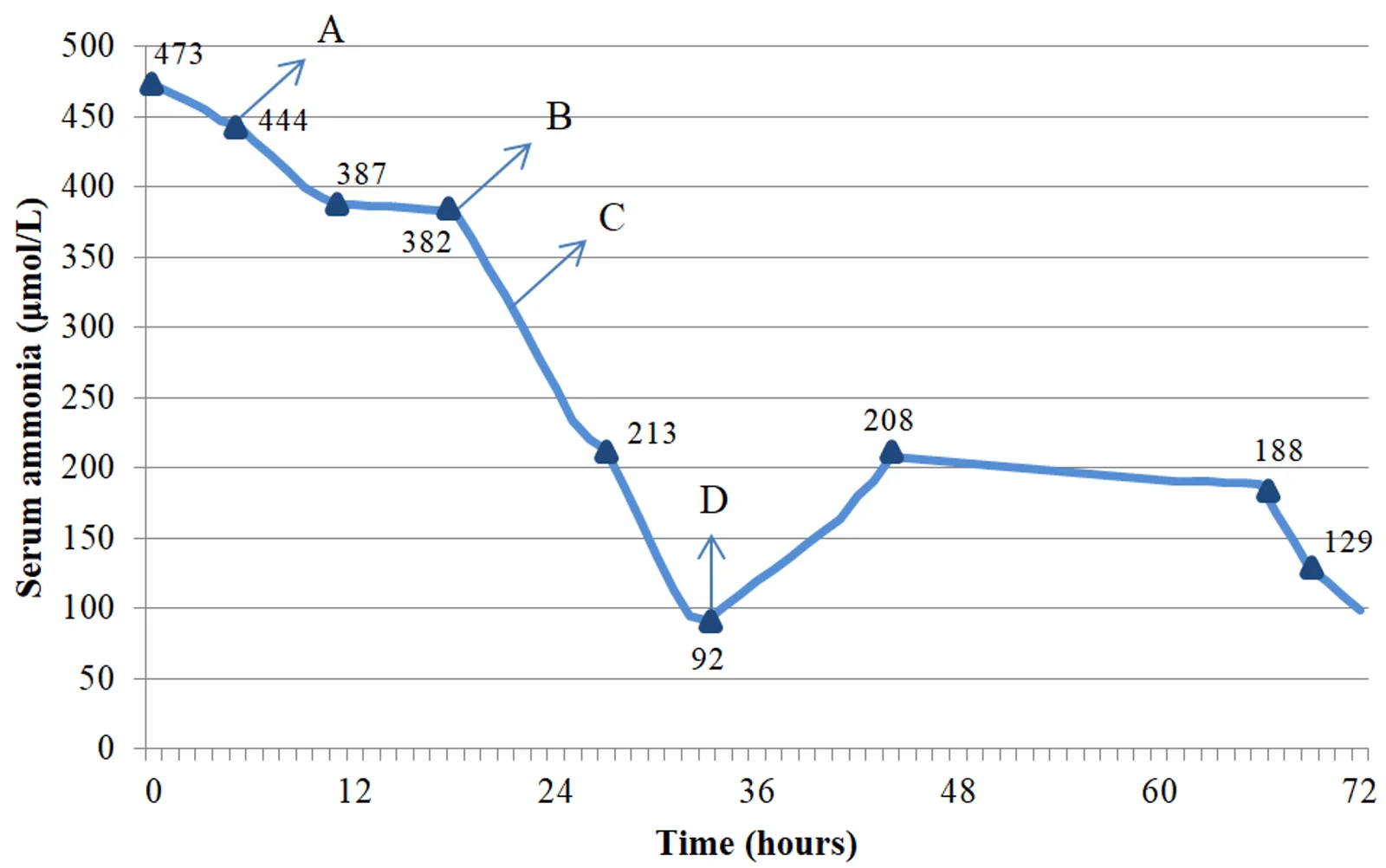
Serum ammonia levels from admission to hyperammonemia resolution A: first CRRT session starts with standard protocol, B: first CRRT session ends, C: second CRRT session starts with intensified protocol, D: second CRRT session ends.

## Discussion

Neonatal-onset type 1 citrullinemia represents the most severe form of the disease and is characterized by life-threatening hyperammonemia [1-5]. Because prolonged exposure to elevated ammonia rapidly results in cerebral injury and death, timely reduction of plasma ammonia is central to successful management [1,6,7]. In patients receiving conservative therapy with plasma ammonia concentrations between 300 and 500 μmol/L accompanied by moderate to severe encephalopathy, coma, or seizures, urgent initiation of kidney replacement therapy is indicated [8]. Intermittent hemodialysis achieves the highest instantaneous ammonia clearance [1,8,9]. However, its application in neonates remains limited by technical constraints and the possibility of post-dialysis ammonia rebound, often necessitating subsequent CKRT [8,9]. Consequently, continuous modalities frequently represent the only practical treatment option in institutions without neonatal-specific dialysis platforms, as was the case in our center.

Our case illustrates that conventional CKRT prescriptions may provide insufficient ammonia clearance in neonates with severe hyperammonemia, whereas treatment intensification can dramatically improve ammonia clearance, resulting in rapid neurological recovery. Several recent reports have demonstrated that intensified CKRT protocols substantially increase ammonia clearance by combining higher blood flow rates (Qb 8-10 mL/kg/min vs. the conventional 3-5 mL/kg/min), higher dialysate or replacement fluid flow rates (up to 8,000 mL/h/1.73 m² vs. approximately 2,000 mL/h/1.73 m² with standard prescriptions), and, in selected cases, larger membrane surface areas than those conventionally recommended for neonatal patients [2,7,10]. Nevertheless, standard CKRT may still have an important role following the initial rapid reduction in plasma ammonia concentrations by maintaining plasma ammonia concentrations within the target range of 100-200 μmol/L in patients whose metabolic control cannot be achieved with conservative therapy alone [7].

The physicochemical characteristics of ammonia, including its small molecular size and negligible protein binding, favor diffusive rather than convective removal, supporting the use of hemodialysis-based techniques when rapid clearance is required [7,8]. Consequently, CVVHD was selected during the intensified treatment phase and resulted in excellent biochemical and clinical responses. This observation further supports the preferential use of diffusive clearance strategies when rapid ammonia removal is required in neonatal hyperammonemia.

A technical challenge in centers without neonatal dialysis platforms is the relatively large extracorporeal circuit volume, which may contribute to hemodynamic instability in very small infants. To minimize this risk, priming the extracorporeal circuit with packed red blood cells before treatment initiation is recommended [2,7,10], as was done in our patient. Importantly, no hemodynamic instability occurred during circuit connection or throughout either CKRT session in our patient despite the relatively large extracorporeal circuit volume.

Intensified CKRT may be associated with electrolyte disturbances; therefore, plasma electrolyte concentrations should be monitored frequently, ideally every four hours, to allow for timely detection and correction [10]. In our patient, hypokalemia, hypomagnesemia, and hypophosphatemia were promptly recognized through close monitoring and effectively corrected.

## Conclusions

Alongside conventional medical management, intensified CKRT represents a feasible and highly effective strategy for the rapid control of severe hyperammonemia in neonates with type 1 citrullinemia, serving as a bridge to liver transplantation, the preferred curative treatment. In our patient, escalation from a conventional to an intensified prescription resulted in rapid ammonia clearance and prompt neurological improvement without treatment-related hemodynamic complications. Although evidence remains limited because of the rarity of neonatal urea cycle disorders, the accumulation of multicenter experience will be essential to define the optimal CKRT prescription and improve long-term neurological outcomes. Early diagnosis, together with timely initiation and optimization of CKRT, may prevent severe neurological sequelae in affected neonates.

## References

[1] Quinonez SC, Lee KN (2026). GeneReviews®. GeneReviews® [Internet].

[2] Tosun D, Akcay N, Menentoglu EM, Sevketoglu E, Salihoglu O (2022). Newborn treated with continuous renal replacement therapy for citrulinemia-type 1. Sanamed.

[3] Rathore AS, Shravya LG, Seth D (2022). A rare case of type 1 citrullinemia in newborn. Int J Contemp Pediatr.

[4] Biyani G, Azad SM, Guha S, Kapoor R (2017). Citrullinemia type 1: a rare case. Ann Int Med Dent Res.

[5] Deng L, Liu Y, Chen K, Qiu J, Chang L, Xia J, Wang Y (2025). Case Report: From coma to genetic insights: identification of a novel pathogenic variant in Chinese neonatal CTLN1. Front Pediatr.

[6] Häberle J, Burlina A, Chakrapani A (2019). Suggested guidelines for the diagnosis and management of urea cycle disorders: first revision. J Inherit Metab Dis.

[7] Spinale JM, Laskin BL, Sondheimer N, Swartz SJ, Goldstein SL (2013). High-dose continuous renal replacement therapy for neonatal hyperammonemia. Pediatr Nephrol.

[8] Raina M, Doshi K, Myneni A, Tibrewal A, Gillen M, Hu J, Bunchman TE (2025). Inborn errors of metabolism in neonates and pediatrics on varying dialysis modalities: a systematic review and meta-analysis. Pediatr Nephrol.

[9] McBryde KD, Kershaw DB, Bunchman TE, Maxvold NJ, Mottes TA, Kudelka TL, Brophy PD (2006). Renal replacement therapy in the treatment of confirmed or suspected inborn errors of metabolism. J Pediatr.

[10] Markham C, Williams C, Miller C, Grange DK, Davis TK, Remy KE (2021). Continuous renal replacement therapy for two neonates with hyperammonemia. Front Pediatr.

